# Identification and characterization of pathogens causing saffron corm rot in China

**DOI:** 10.3389/fmicb.2023.1188376

**Published:** 2023-06-09

**Authors:** Tingdan Ren, Dejiang Dai, Miao Yu, Tao Li, Chuanqing Zhang

**Affiliations:** ^1^College of Modern Agriculture, Zhejiang Agriculture and Forest University, Hangzhou, China; ^2^Station for the Plant Protection, Quarantine and Control of Agrochemicals of Zhejiang Province, Hangzhou, China

**Keywords:** *Crocus sativus* corm rot, amplicon sequencing (16SITS), microbial diversity, pathogen identification, occurrence regularity

## Abstract

Corm rot is the most important disease of saffron, for which fungi from several genus such as *Fusarium* spp. *Penicillium* spp. and *Botrytis* spp., have been previously reported to be the pathogens. In this research, we used a combination of amplicon sequencing and traditional isolation methods to identify the causal agents, main infection source. The diversity of microbial communities in diseased saffron corms and soil decreased significantly compared with healthy corms and soil. The contents of *Penicillium* and *Botrytis* in healthy and diseased corms were similarly high, indicating that them were not directly related to the occurrence of corm rot. But the relative abundance of *Fusarium*, *Cadophora* and *Fusicolla* were significantly higher in the diseased corms than healthy ones. The abundance of *Fusarium* increased, while the abundance of *Oidiodendron*, *Paraphaeosphaeria* and the endophytic beneficial bacteria *Pseudomonas* decreased, which may relate to the occurrence of the disease. The co-occurrence network diagram showed that the correlation between fungal and bacterial communities was mainly positive. Plant pathogens were relatively abundant in the diseased soil, according to functional gene prediction. At the same time, we also collected 100 diseased corms from the fields in Jiande, where is known as the “hometown of saffron.” All isolated pathogenic strains were identified as *Fusarium oxysporum* through morphological observation and phylogenetic tree analysis of ITS, *Tef-1α* and *β-tubulin*. To better clarify the biological characteristics of *F*. *oxysporum*, we cultured the isolates at different temperatures and pH values. The optimum temperature for mycelial growth and sporulation was 25°C, pH 6，carbon sources sorbitol and nitrogen sources, peptone. In short, our results suggests that *F. oxysporum* was the pathogen causing corm rot in Jiande and corms other than soils are the main primary infection source. These new understanding of saffron corm rot will provide the theoretical basis for its better and efficiently management.

## Introduction

Saffron (*Crocus sativus*) is a perennial herb of the genus *Crocus* L. in Iridaceae (Liu et al., 2022). In China, saffron is well known as a rare Chinese herbal medicine, affecting the activation of blood circulation and dispersion of blood stasis, cooling blood, causing detoxification, relieving depression, facilitating the improvement of immunity, and preventing the occurrence of cardiovascular disease (Jia et al., 2022). It is also a precious seasoning agent, natural pigment, spice and dye abroad. Saffron originates in Mediterranean countries, such as Iran, Spain and Greece, where are currently the largest planting regions in the world (Bahrami et al., 2020). The birthplace of saffron cultivation in China is Hangzhou. Sandu Town of Jiande City (29.54 N, 119.57E) in Hangzhou is the main production area of saffron in China, where is known as the “hometown of saffron in China” and provides 80% yields of saffron (Chen, 2018). Saffron corm rot (SCR) is the most serious disease threatening its production. In the corm storage and planting periods, the rate of diseased corms can be as high as 70%, decreasing the quality of corms and thrums and resulting in large economic losses (Hu et al., 2021).

The typical symptoms of SCR in the fields are yellow withered leaves on the ground and atrophied rotting corms underground. About 11 genera of fungi generally collected from regions other than Sandu have been reported to be correlated with SCR. Among them, *Fusarium*, *Aspergillus*, and *Penicillium* were frequently detected (Zhang et al., 2009; Ahrazem et al., 2010; Zhao, 2014; Wei et al., 2021). SCR is considered as a typic soil-borne disease until present and therefore the “saffron-rice” rotation was adopted as the main technique for its management. The “drought (saffron) -rice” rotation has been suggested to be the most beneficial technique for the prevention and control of soil-borne diseases (Wang et al., 2021). In Jiande, when the thrums of saffron are picked and the corms are stored indoors, rice is planted in the fields, and saffron is replanted after the rice harvest (Deng et al., 2019). However, the occurrence of SCR is still aggravating.

Plant pathogens have traditionally been discovered and identified based on culture-dependent methods. However, due to the influence of surface sterilization methods, culture media, culture conditions (Dissanayake et al., 2018) and other factors, traditional isolation and culture results cannot fully reflect the number and role of microorganisms or pathogens in plants and soil. Amplicon sequencing based on high-throughput sequencing is widely used to study leaf/rhizosphere microbial community and diversity (Luo et al., 2019; Liu et al., 2021; Masenya et al., 2021). Some studies using amplicon technology have reported that asymptomatic leaves have a higher microbial diversity and richness than symptomatic leaves (Xiang et al., 2022). This technology can unravel the causalities between microbiome assembly and disease onset. Changes in the rhizosphere microbiome composition can predict whether plants remain healthy or become infected by the pathogen (Gu et al., 2022). Therefore, the combination of amplicon analysis and traditional culture-dependent methods can perhaps more comprehensively reflect the composition of phyllosphere/rhizosphere microbial communities (Paloma et al., 2018). Therefore, the objectives of this study were to: (i) identify and characterize the pathogen of SCR by both traditional tissue isolation and phylogenetic analysis and (ii) investigate the main infection source of SCR. These results will change our view of understanding SCR and provide reasonable information and help for management of SCR.

## Materials and methods

### Analysis of microbial diversity in corms and rhizosphere soil

On 16 April, 2021, saffron corms and rhizosphere soil samples were collected from saffron fields in Sandu Town of Jiande (Table 1). For each treatment, 10 were healthy (aboveground and underground healthy) and 10 were visibly rotted and odorous (underground rot). The 10 corms were cut into small pieces, mixed, weighed 10 g into a large centrifuge tube. This was repeated three times for each treatment. Meanwhile, rhizosphere soil (15–20 cm depth) was dug from the collected corms of saffron, and the root system and other impurities were removed. Ten rhizosphere soils were mixed into one soil sample (10 g) with three replicates. These samples were sequenced by the second-generation sequencing to analyze fungal and bacterial community on the Illumina Miseq/Novaseq Platform (Illumina, San Diego, United States) at Baimaike Biotechnology Co., LTD (Bokulich et al., 2013; Xiang et al., 2022). Corms or soils, total genome DNA from samples was extracted according to manufacturer’s protocols. The sequencing library was constructed using a MetaVX Library Preparation Kit (Beijing Baimaike Biotechnology Co., LTD, Nanjing, China). Briefly, 20–50 ng of DNA was used to generate amplicons that cover ITS rRNA region of Fungal organisms and V3 and V4 hypervariable regions of the 16 s rRNA gene of bacteria. Automated cluster generation and 250/300 paired-end sequencing with dual reads were performed according to the manufacturer’s instructions.

**Table 1:** Sample and analysis content of microbial diversity in corms and rhizosphere soil of saffron.

Sample	Sample name	Analysis content
Rhizosphere soil of healthy corm	HS	Fungi ITS1/Bacteria 16S rDNA V3 + V4
Rhizosphere soil of diseased corm	US	Fungi ITS1/Bacteria 16S rDNA V3 + V4
Healthy corm	HC	Endophytic fungi ITS1/Endophytic bacteria 16 s V3 + V4
Rotted corm	RC	Endophytic fungi ITS1/Endophytic bacteria 16 s V3 + V4

### Fungal isolation

In April 2021, one hundred diseased saffron corms with typical symptoms of SCR were collected from saffron planting bases in Sandu Town, Jiande City, Zhejiang Province. The diseased corms were washed with running tap water and cut into 5 × 5 mm pieces using sterilized scissors. Subsequently, the tissue pieces were soaked in 75% alcohol for 30 s and in 3% sodium hypochlorite solution for 2 min, then rinsed with sterile distilled water three times, and dried on sterile filter paper. Each tissue piece was placed on a plate containing potato dextrose agar (PDA) medium supplemented with kanamycin sulfate (100 mg/L) and streptomycin sulfate (100 mg/L) and incubated at 25°C. After incubation for 3 to 5 days, mycelia were transferred to a new PDA plate from the margin of the colonies (Phoulivong et al., 2010; Noman et al., 2018). Subsequently, single spore isolation was conducted, and single-conidium isolates were stored on PDA slants at 4°C.

### Pathogenicity testing

All isolates were cultured on a PDA medium for 5 days at 25°C. The spore suspensions were prepared following a previous study (Gale et al., 2005). In detail, five mycelial plugs (diameter of 5 mm) taken from the colony margin were transferred to a 100 mL PD liquid medium and shaken at 160 r/min and 25°C to induce conidia production. After 5 days, spores were collected and adjusted to 1.0 × 10^6^ conidia/mL using a hemocytometer (Hu et al., 2022).

Before inoculation, healthy corms were washed with water and disinfected with 75% alcohol by wiping the surface. Then corms had been washed properly with sterilized distilled water and natural air-dry. Each corm was inoculated with 10 μL of a conidial suspension. The negative control was inoculated with sterile water, and three corms were inoculated for each isolate. The inoculated corms were then placed in a chamber at 80% humidity and a 12 h light/dark cycle for 7 days at 25°C. The pathogens were reisolated from the diseased corms to complete Koch’s postulates (Chen et al., 2020).

### Morphological characterization

Morphological characterizations were performed according to the methods described by Cai et al. (2009). The mycelial plugs (5 mm in diameter) extracted from the edge of the purified isolates were transferred to a new PDA plate central and cultured at 25°C. Five days later, colony morphology and color were observed and photographed, and the morphology and size of the conidia were observed and recorded under a light microscope (Carl Zeiss Microscopy GmbH, Gottingen, Germany).

To observe the conidial fructification and chlamydospores, a carnation leaf culture medium (CLA) was used (Qiu et al., 2021). Carnation leaves were sterilized with 70% alcohol and 2% sodium hypochlorite, cut into pieces (10 mm^2^), placed in the oven for drying, and placed on the surface of the water agar. Seven to 10 leaf pieces were added to a single 90 mm Petri dish, and the mycelial plugs were placed close to the. After 5 days of growth on CLA medium, large and small conidia, conidial fructification, and chlamydospores were observed under a light microscope.

### Molecular identification and phylogenetic analysis

Based on morphological identification, ITS*, Tef-1α* and *β-tubulin* genes were used for molecular identification (Britz et al., 1998; Rahjoo et al., 2008; Crous et al., 2021). The isolates were cultured on PDA until the colony covered the plate, and the DNA of each isolate was extracted using a fungus genomic DNA rapid extraction kit (B518229-0100; Sangon Biotech, Shanghai, China). The ITS genes of the isolates were amplified using universal primers ITS1 (5′-TCCGTAGGTGAACCTGCGG-3′) /ITS4 (5’-TCCTCCGCTTATTGATATGC-3′). Partial fragments of *Tef-1α* and *β-tubulin* genes were amplified using polymerase chain reaction (PCR) with specific primers *EF-1* (5′-ATGGGTAAGGAAGACAAGAC-3′)/*EF-2* (5’-GGAAGTACCAGTGATCATG-3′) and *T1* (5′-AACATGCGTGAGATTGTAAGT-3′)*/T22* (5’-TCTGGATGTTGTTGGGAATCC-3′), respectively (Amata et al., 2010; O’Donnell et al., 2010).

The PCR system was as follows: 25 μL Taq PCR Master Mix, 1 μL upstream primer and 1 μL downstream primer, 2 μL template DNA and 21 μL ddH_2_O (i.e., 50 μL total volume). The PCR amplification procedure was as follows: 95°C for 5 min; 35 cycles of 95°C for 30 s, 55°C for 1 min, 72°C for 50 s; and 72°C for 10 min. The PCR products were identified on 1.0% agarose gel at 254 nm (UV) and further sequenced by Sangon Biotech.

The sequencing results were submitted to the NCBI database^1^ for comparison and identification. The ITS, *Tef-1α* and *β-tubulin* standard gene sequences were downloaded from GenBank. *F. proliferatum* was also downloaded as the outgroup due to its distant genetic relationship with the isolates. After all sequences were integrated, MEGA 6.0 was used for sequence alignment analysis, and PAUP 4.0 was used for format conversion after cutting. The MrMT-gui model calculation was used to identify the best-matching model. Finally, MrBayes 3.2.6 was used to construct the phylogenetic tree (Hu et al., 2022).

### Evaluation of the effects of temperature and pH on growth and sporulation

Three strains of *F*. *oxysporum* were randomly selected. Five-day-old mycelial plugs were extracted with a sterile 5 mm diameter hole puncher and transferred to the center of the PDA medium. They were incubated at 10°C, 15°C, 20°C, 25°C, 30°C, and 35°C (Ren et al., 2022; Zhou et al., 2022) in the dark for 5 days. The colony diameter was measured in two approximately perpendicular directions, and conidia were collected from the plates and counted with a hemocytometer (Li et al., 2022). There were three replications for each isolate and the experiment was conducted three times.

Mycelial plugs with a diameter of 5 mm were used to inoculate the PDA medium with pH 4, 6, 8, and 10 and incubated at 25°C at a constant temperature for 5 days (Huo et al., 2022). The colony diameter was measured in two approximately perpendicular directions. The spore suspension was prepared, and the spores were counted under a microscope (Wang et al., 2022). Each treatment contained three petri dishes, and the experiment was repeated three times.

### Effects of different carbon and nitrogen sources on growth and sporulation

Czapek (Krzyśko-Lupicka et al., 1997) medium was used as the base medium. The mycelial plugs (0.5 cm in diameter) were inoculated on the plates with sucrose, cellulose, sorbitol, galactose, fructose, maltose, glucose and soluble starch as different carbon sources, or with cysteine, ammonium sulfate, ammonium nitrate, sodium nitrate, beef extract, glycine, yeast extract and peptone as different nitrogen sources. Czapek plates without carbon or nitrogen source were used as control, and each treatment was repeated three times. After incubation at 25°C in a constant temperature incubator, the colony diameter was recorded, and conidia were collected from the plates and counted with a hemocytometer, respectively (Li et al., 2022).

### Data processing and statistical analysis

The QIIME data analysis package was used for ITS rRNA and 16S rRNA data analysis. Quality filtering on joined sequences was performed and sequence which did not fulfill the following criteria were discarded: sequence length < 200 bp, no ambiguous bases, mean quality score ≥ 20 (Gao et al., 2021). Then the sequences were compared with the reference database (RDP Gold database) using UCHIME algorithm to detect chimeric sequence, and then the chimeric sequences were removed. The effective sequences were used in the final analysis. Sequences were grouped into operational taxonomic units (OTUs) using the clustering program VSEARCH(1.9.6)against the UNITE ITS database^2^ and 16S rRNA reference database Silva pre-clustered at 97% sequence identity (Wei et al., 2018). Then using RDP classifier (Ribosomal Database Program) Bayesian algorithm on the 97% similarity level OTU representative sequence analysis of taxonomic RDP Classifier, and, respectively, in the following taxonomic levels: domain, and kingdom, phylum, class, order, family, genus, species community composition was analyzed, and the confidence reading value was 0.7. Use Mothur pieces on the 97% similarity level diversity analysis (Lu et al., 2017; Paloma et al., 2018).

Based on OTU analysis results are obtained, using the method of random sampling sample sequences is flat, calculate Shannon, Chao1 alpha diversity index, community species Abundance and diversity of Rarefaction curves and Rank—Abundance graph can also reflect the species richness and evenness (Paulson et al., 2013).

A network diagram is a form of correlation analysis. According to the abundance and variation of each species in each sample, the Spearman correlation analysis was performed, and data with a correlation greater than 0.1 and a *value of p* less than 0.05 were selected to construct the correlation network, can directly see the influence of different environmental factors on the microbial adaptation, and an environment of mutual advantage of the dominant species, close interactions of species, the dominant species and the species group tend to maintain the environment of the microbial community structure and function stability plays a unique and important role (Grady et al., 2019).

All visualizations were made by R software (version 4.0.2). All data were analyzed with the Excel and SPSS software program (version 22.0 for Windows).

## Results

### Disease symptoms

**Figure 1:**
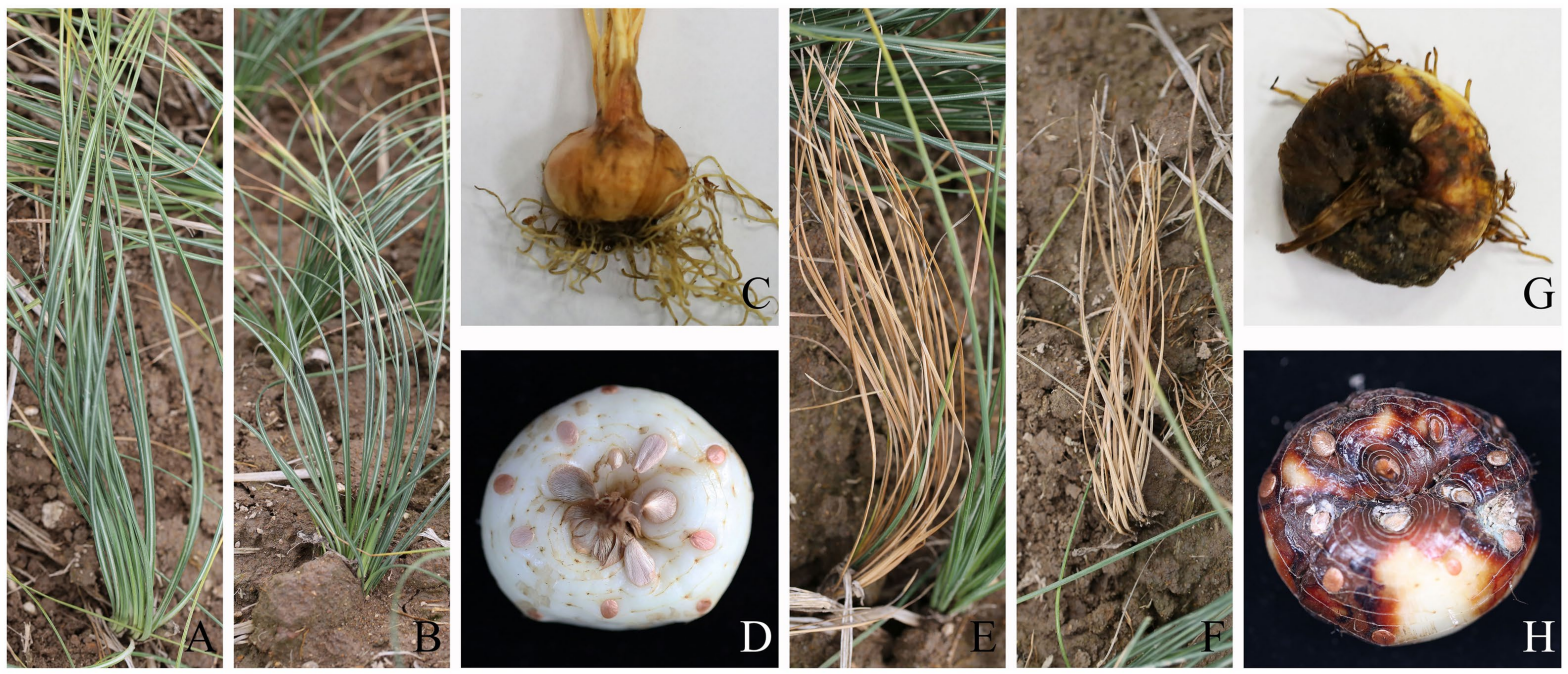
Field symptoms of saffron corm rot disease in aboveground parts (**A,B** for healthy and **E,F** for diseased) and corms (**C,D** for healthy and **G,H** for diseased).

The typical symptoms of saffron rot were plant withering, leaf yellowing and wilting on the ground, and diseased corms were tawny to black, decayed and soft in the field (Figure 1). During the storage period, some brown to reddish brown spots occurred on the surface of diseased corms, and the whole corm gradually rotted.

### Microbial community composition of healthy and diseased corms and rhizosphere soils

**Figure 2:**
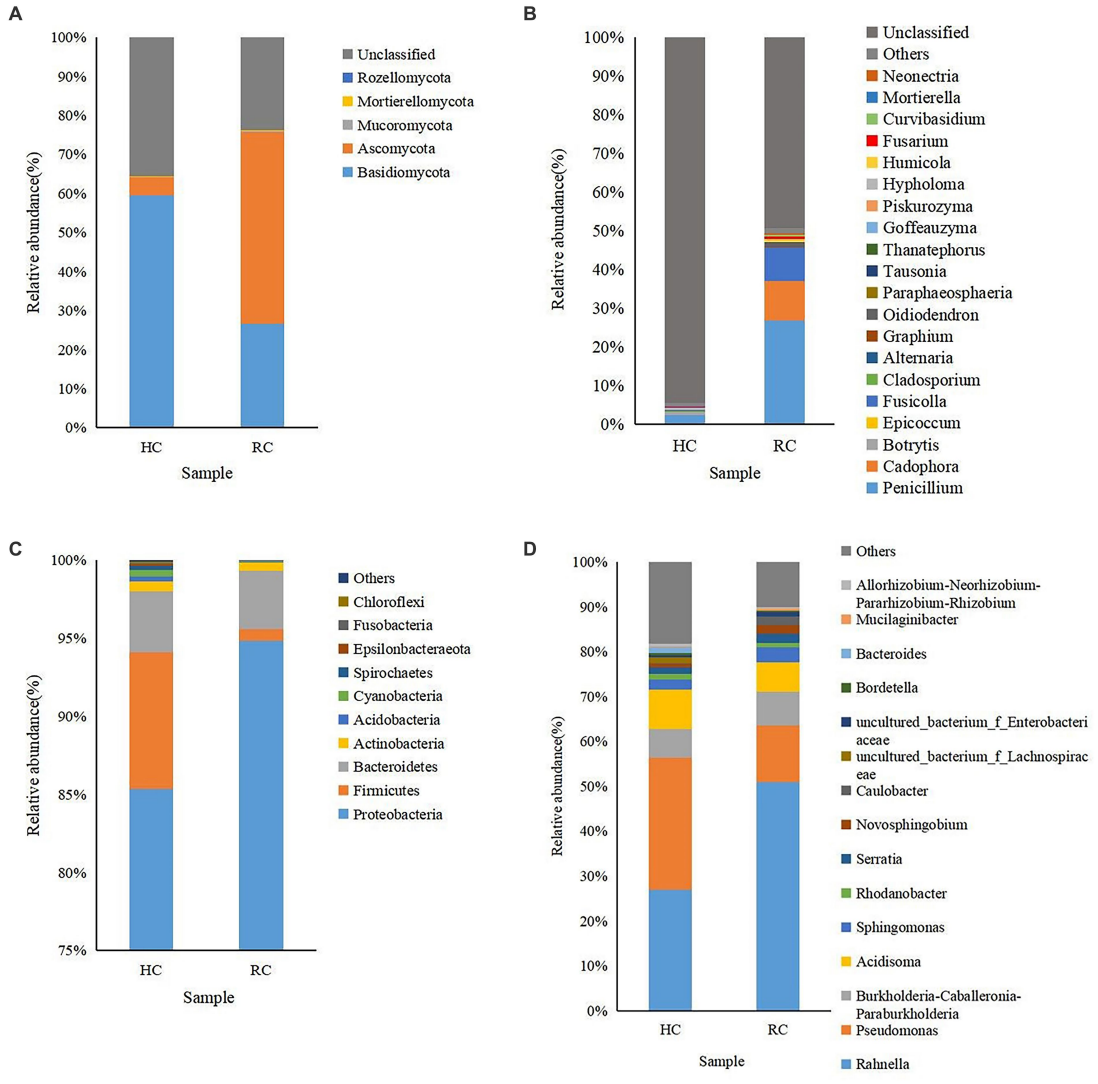
Microbial community composition of different corms (HC, healthy corm; RC, rotted corm) at the phylum (**A** for fungi and **C** for bacteria) and genus (**B** for fungi and **D** for bacteria) levels. The relative abundance of phyla or genera making up less than 0.01% was classified as “Others.”

A rich microbial community was observed in both healthy and diseased corms. In fungal communities, the relative abundance of Basidiomycota gradually decreased from healthy to rotted corms at the phylum level, while the relative abundance of Ascomycota was the opposite (Figure 2A). The abundance of *Penicillium* was significantly higher in wilting and rotting corms than in healthy corms. In addition, the relative abundance of *Cadophora*, *Fusicolla*, *Oidiodendron,* and *Fusarium* increased in rotting corms (Figure 2B).

Aside from “others,” the dominant identified bacterial phyla in all types of corms were Proteobacteria, Firmicutes and Bacteroidetes, with relative abundances ranging from 0.74% to 94.84%. The relative abundance of Proteobacteria increased significantly in the diseased corms (Figure 2C). Most of the top 20 most abundant bacterial genera were non-pathogenic. The relative abundance of *Rahnella*, *Pseudomonas* and *Acidisoma* was relatively high. The abundance of *Rahnella* in diseased corms increased significantly, while that of *Pseudomonas* decreased significantly, accounting for only 0.45%–12.55% (Figure 2D).

**Figure 3:**
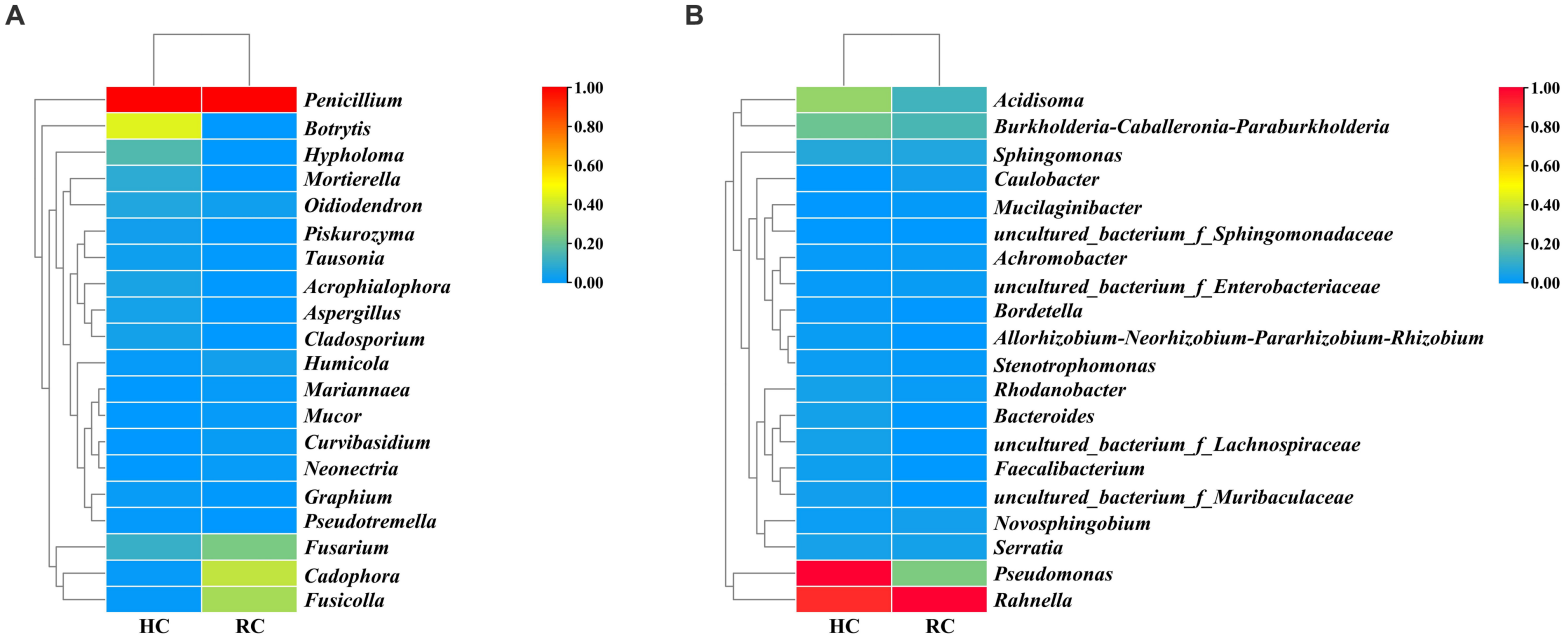
Heat map of fungal **(A)** and bacterial **(B)** species richness clustering in healthy and rotted corms.

Cluster analysis was performed according to the composition of each sample at the genus level (Stone et al., 2018), and the relative abundance of the top 20 genera in each sample was presented in heat maps (Figure 3). In both healthy and rotted corms, *Penicillium* was the most abundant fungal genus, and *Rahnella* was the most abundant bacterial genus. Compared to healthy corms, the relative abundance of *Botrytis*, *Acidisoma* and *Pseudomonas* decreased, while the relative abundance of *Fusarium*, *Cadophora* and *Fusicolla* increased in rotted corms. These results suggest that the microbial community composition was changed by the development of saffron corm rot disease. The community composition of diseased corms was significantly different.

**Figure 4:**
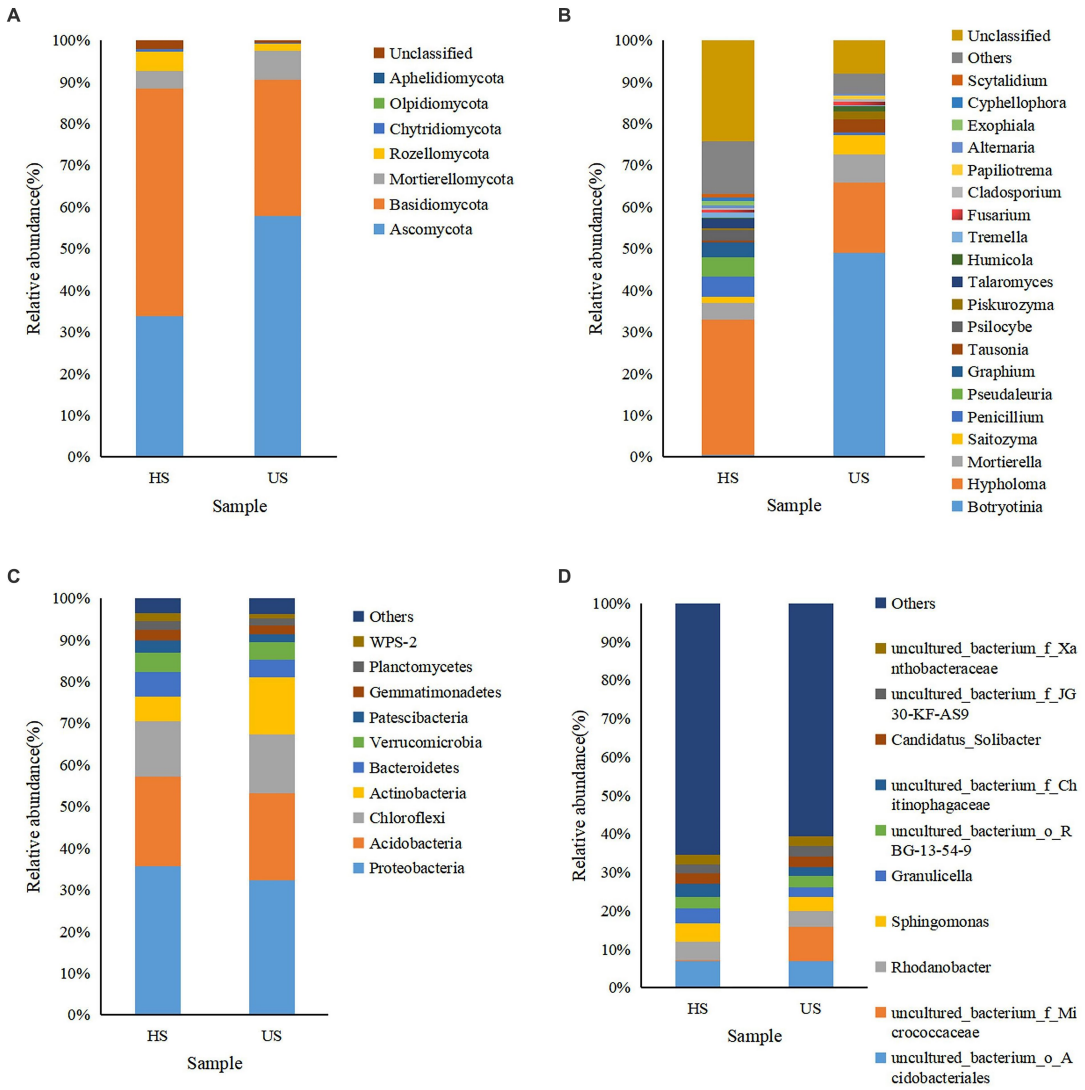
Microbial community composition of different rhizosphere soil samples (HS, healthy soil; US, unhealthy soil) at the phylum (**A** for fungi and **C** for bacteria) and genus (**B** for fungi and **D** for bacteria) levels. The relative abundance of phyla or genera that made up less than 0.01% was classified as “Others.”

Based on weighted UniFrac analysis (Laforest-Lapointe et al., 2017), the fungal community in two soil samples was distributed in at least seven defined phyla (Figure 4). The dominant fungi in the two soil samples were Basidiomycota and Ascomycota at the phylum level, and the relative abundances of these fungi were quite different between the two soil samples. For Ascomycota, it was 33.72% in healthy soil samples and increased to 57.86% in diseased soil samples, while Basidiomycota decreased from 54.63% in healthy soil samples to 32.63% in diseased soil samples (Figure 4A). At the genus level, *Hypholoma* was the most abundant species in healthy soil samples (32.44%) and decreased to 16.75% in diseased soil. *Botryotinia* was the most abundant genus in diseased soil samples, while it was much lower in healthy soil (0.41%). Unexpectedly, the relative abundance of *Fusarium* was approximately the same in the two soil samples, at 0.59% and 0.74% for diseased soil and healthy soil, respectively.

In the bacterial community, Proteobacteria, Acidobacteria, Chloroflexi, and Actinobacteria were relatively abundant in the two soil samples at the phylum level. However, the relative abundance of Actinobacteria increased from 5.84% in healthy soil to 13.64% in unhealthy soil. At the genus level, the uncultured bacterium *f Micrococcaceae* showed the highest relative abundance in the diseased soil sample, while it was very low in the healthy soil sample. *Rhodanobacter*, *Sphingomonas*, *Granulicella* and uncultured bacterium *Chitinophagaceae* were relatively abundant in the healthy soil sample.

### Alpha diversity for the microbial community of healthy and diseased corms and rhizosphere soils

Alpha diversity refers to the diversity including a specific region or ecosystem. The microbial richness including Chao richness estimator (Chao l) and Ace richness estimator (ACE) are commonly measured, reflects the number of species. The microbial diversity can be measured by Shannon-Wiener diversity index (Shannon) and Simpson diversity index (Simpson), reflects the uniform distribution of species in the community.

Alpha diversity indices showed that fungal and bacterial alpha diversity values were different between healthy and diseased corms (Table 2). For the fungal community, the diseased corms exhibited a higher Shannon index and Simpson index and a lower Chao l index and ACE index compared to the healthy corms, but there were no statistically significant differences (*p* = 0.915 > 0.05). For the bacterial community, the diseased corms showed a lower Shannon index, Simpson index, Chao l index and ACE index compared to the healthy corms, but there were no statistically significant differences. There was no significant change in microbial diversity in rotted corms compared with healthy corms.

**Table 2:** Alpha diversity indices of healthy and rotted corm rhizosphere soil microbial communities.

Kingdom	Sample name	OTU numbers	ACE	Chao1	Simpson	Shannon	Goods_coverage
Fungi	HC^X^	142	146.045	145.500	0.602	1.969	1
Fungi	RC	134	143.869	143.231	0.834	3.045	1
Bacteria	HC	463	489.345	529.429	0.876	4.710	0.9997
Bacteria	RC	401	461.895	469.250	0.722	3.433	0.9993
Fungi	HS	539	539.644	541.500	0.880	5.256	0.9999
Fungi	US	357	378.206	380.429	0.722	3.205	0.9995
Bacteria	HS	1,421	1425.497	1430.091	0.992	8.574	0.9996
Bacteria	US	1,418	1421.063	1422.387	0.987	8.419	0.9997

X HC, Healthy corm; RC, Rotted corm; HS, Healthy soil; and US, Unhealthy soil.

As shown in Table 2, bacterial community diversity was much greater than fungal community diversity in rhizosphere soil. For the fungal community, the diseased soil samples had a significantly lower Shannon index, Simpson index, Chao l index and ACE index compared to the healthy soil samples. However, there was no significant difference in the bacterial community.

### Co-occurrence network for microbial communities of healthy and rotted corms

**Figure 5:**
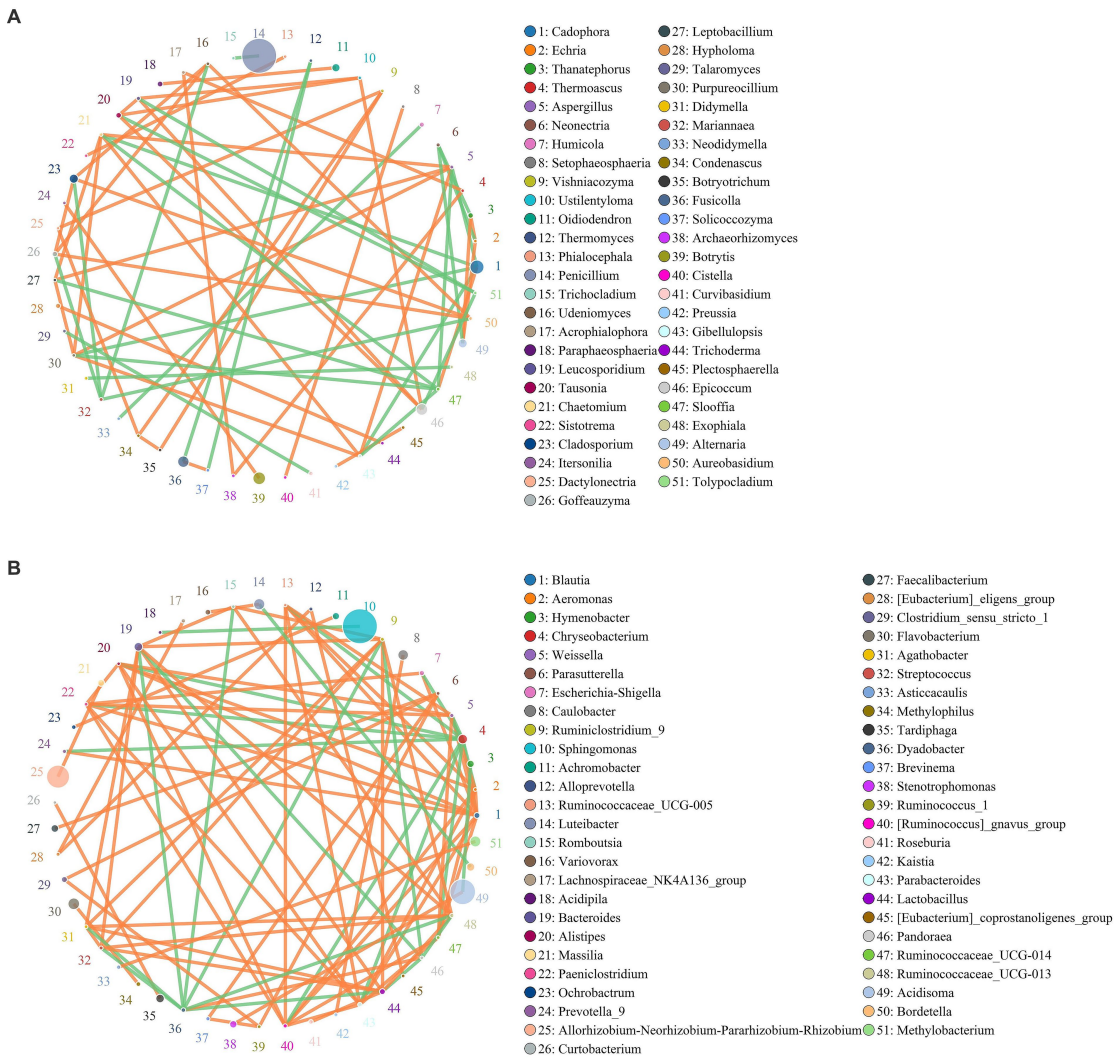
Co-occurrence network for the microbial community of healthy and diseased corms at the genus (**A** for fungi and **B** for bacteria) level. Circles represent species, and the size of the circle represents abundance. The edges represent the correlation between two species. Orange represents a positive correlation, while green represents a negative correlation.

The co-occurrence network diagram showed the top 51 species in terms of correlation strength, and the correlation between fungal and bacterial communities was mainly positive. In the fungal community (Figure 5A), 55 genera had a strong and significant correlation (*p* < 0.05, r > 0.6). There were significant positive correlations between *Botrytis* and *Alternaria*, *Cladosporium* and *Alternaria*, *Alternaria* and *Thanatephorus*, *Oidiodendron* and *Paraphaeosphaeria*, and *Chaetomium* and *Gibellulopsis*. There were 38 genera with strong negative correlations. *Oidiodendron* and *Fusarium*, *Paraphaeosphaeria* and *Fusarium*, and *Cladosporium* and *Mariannaea* had weak negative correlations.

A total of 46 strong negative correlations and 62 strong positive correlations were identified from 71 bacterial genera (Figure 5B). The co-occurring genera were distributed in Bacteroidetes (5.67%, targeted nodes/total nodes), Firmicutes (2.98%), Acidobacteria (0.23%), Patescibacteria (0.24%), Fusobacteria (0.03%), Chloroflexi (0.02%), and other Bacteria (0.20%). *Sphingomonas*, *Acidisoma* and *Allorhizobium*-*Neorhizobium*-*Pararhizobium*-*Rhizobium* were identified as the top three genera based on a high centrality score. A strong negative correlation in the bacterial communities was observed between *Rahnella* and *Bradyrhizobium*, *Sphingomonas* and *Acidipila*, *Acidisoma* and *Hymenobacter*, and *Bacteroides* and *Methylophilus*.

### Prediction of fungal phenotypes in rhizosphere soils

**Figure 6:**
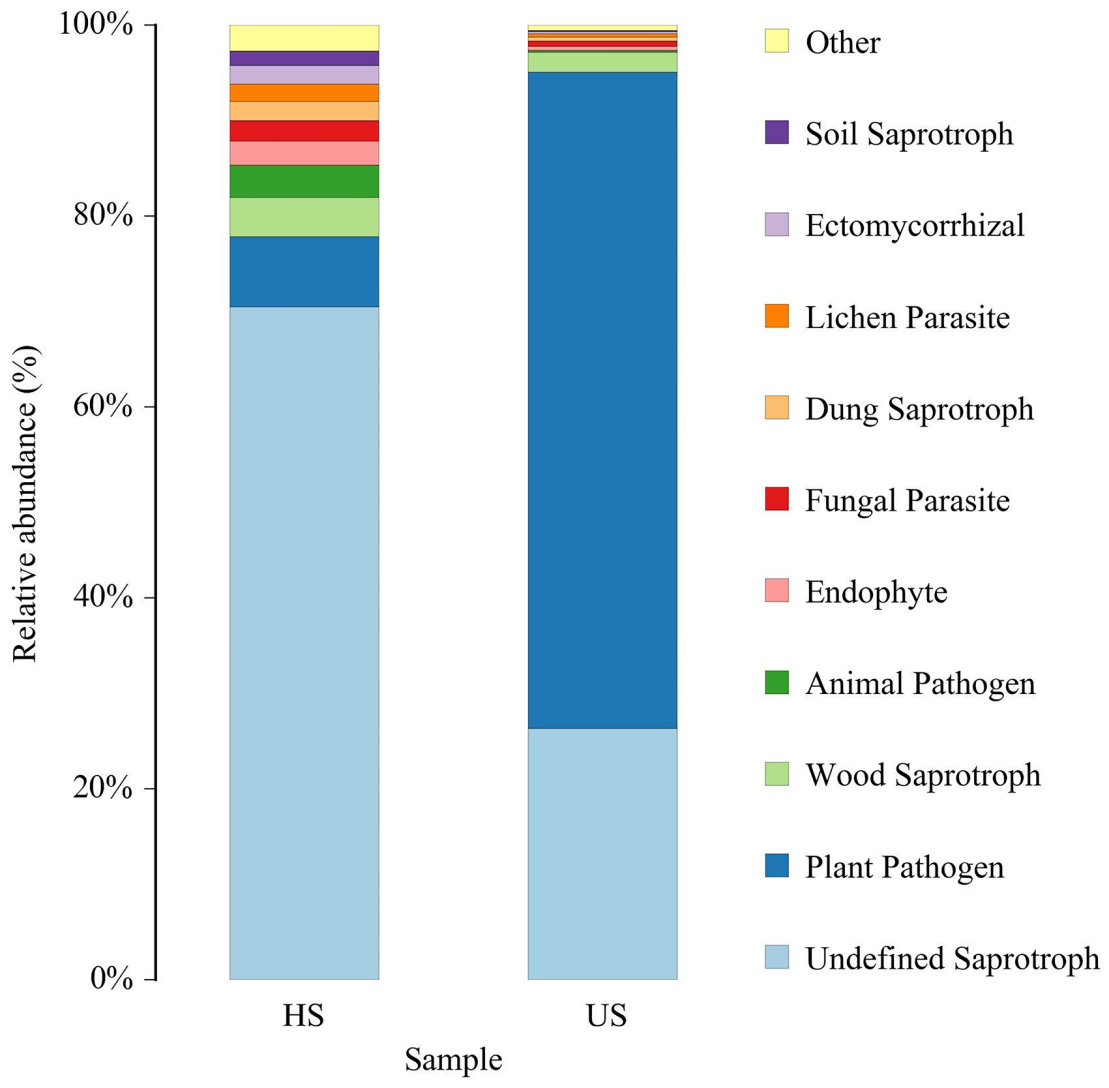
FUNGuild species relative abundance (%) in different rhizosphere soil samples (HS, healthy soil; US, unhealthy soil).

Based on the trophic mode, fungi can be classified into pathotroph, symbiotroph and saprotroph. These three categories are divided into 12 classifications, known as guilds. The Fungi Functional Guild (FUNGuild) annotation platform was used to analyze the ecological function categories of fungi (Nguyen et al., 2016; Chen et al., 2019). The results showed that there were many nutritive fungi in the healthy soil samples (Figure 6), among which the undefined saprotrophic fungi accounted for 75.13%. In the unhealthy soil samples, nutritive fungi were less abundant, while plant pathogens were relatively abundant, accounting for 71.01%.

### Fungal isolation and pathogenicity

**Figure 7:**
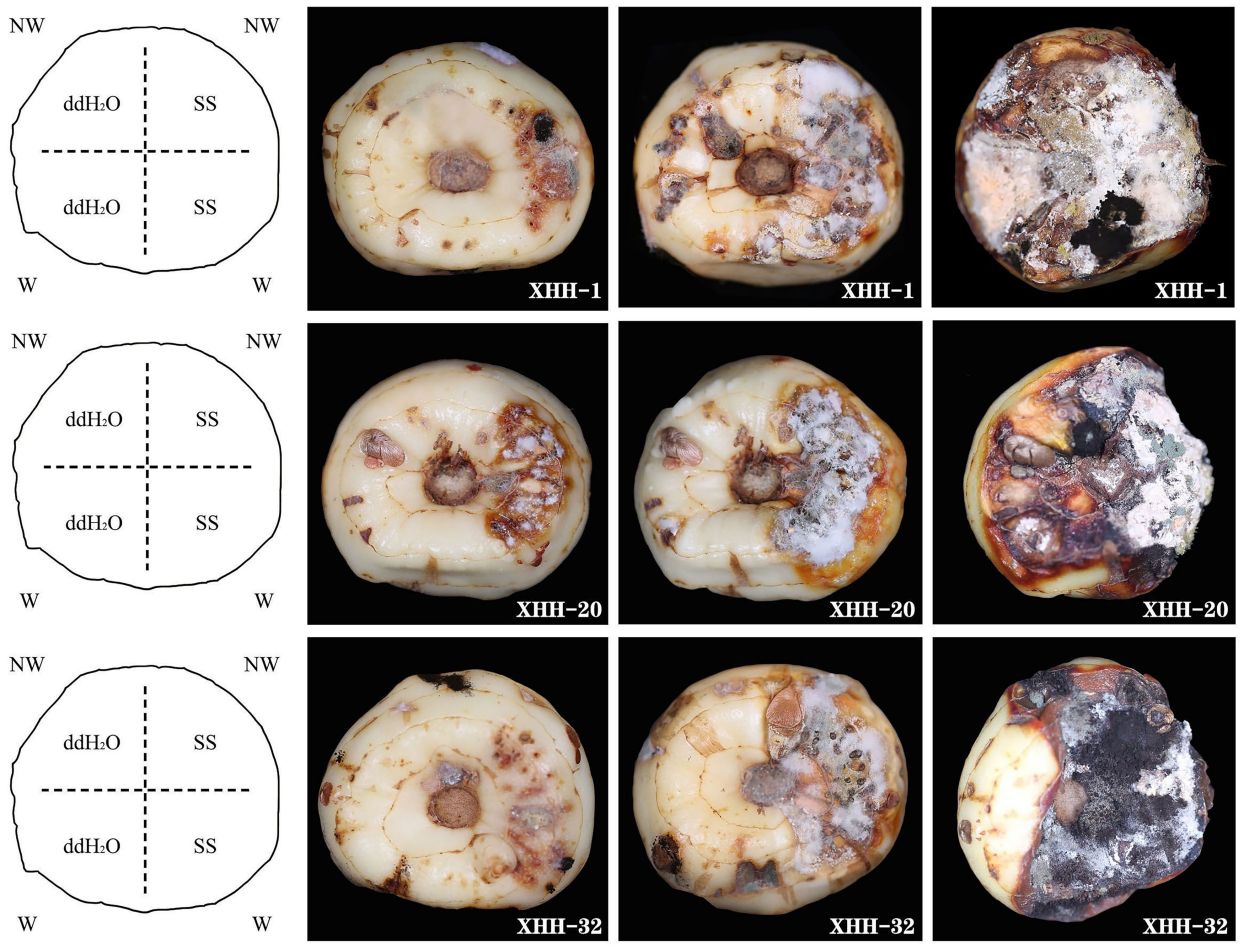
Symptoms on inoculated saffron corms at 5, 10, and 15 days after incubation at 25°C. These isolates were obtained from symptomatic corms. The negative control was inoculated with sterile water. NW, no wound; W, wounded; SS, spore suspension; and ddH_2_O, sterile water.

We isolated 82 strains from 100 diseased saffron corms, were preliminarily identified as *Fusarium*. We tested the pathogenicity of all strains in both unwounded and wounded conditions (Figure 7). Five days after inoculation, disease spots began to appear on the surface of the corms and with white hyphae in the wounded corms. After 10 days, the mycelium expanded and extended into the interior of the corm, accompanied by other epiphytic fungi on the surface. After 15 days, the entire corm shrank, and the spot turned brown. The wounded area gradually decayed and disappeared. The negative control inoculated with sterile water showed no disease when it was wounded or not wounded 15 days after inoculation. After inoculation with the pathogen spore solution, red-brown to brown lesions appeared and gradually spread, and after wounding, the pathogenicity of the saffron corms increased significantly.

### Morphological characteristics

**Figure 8:**
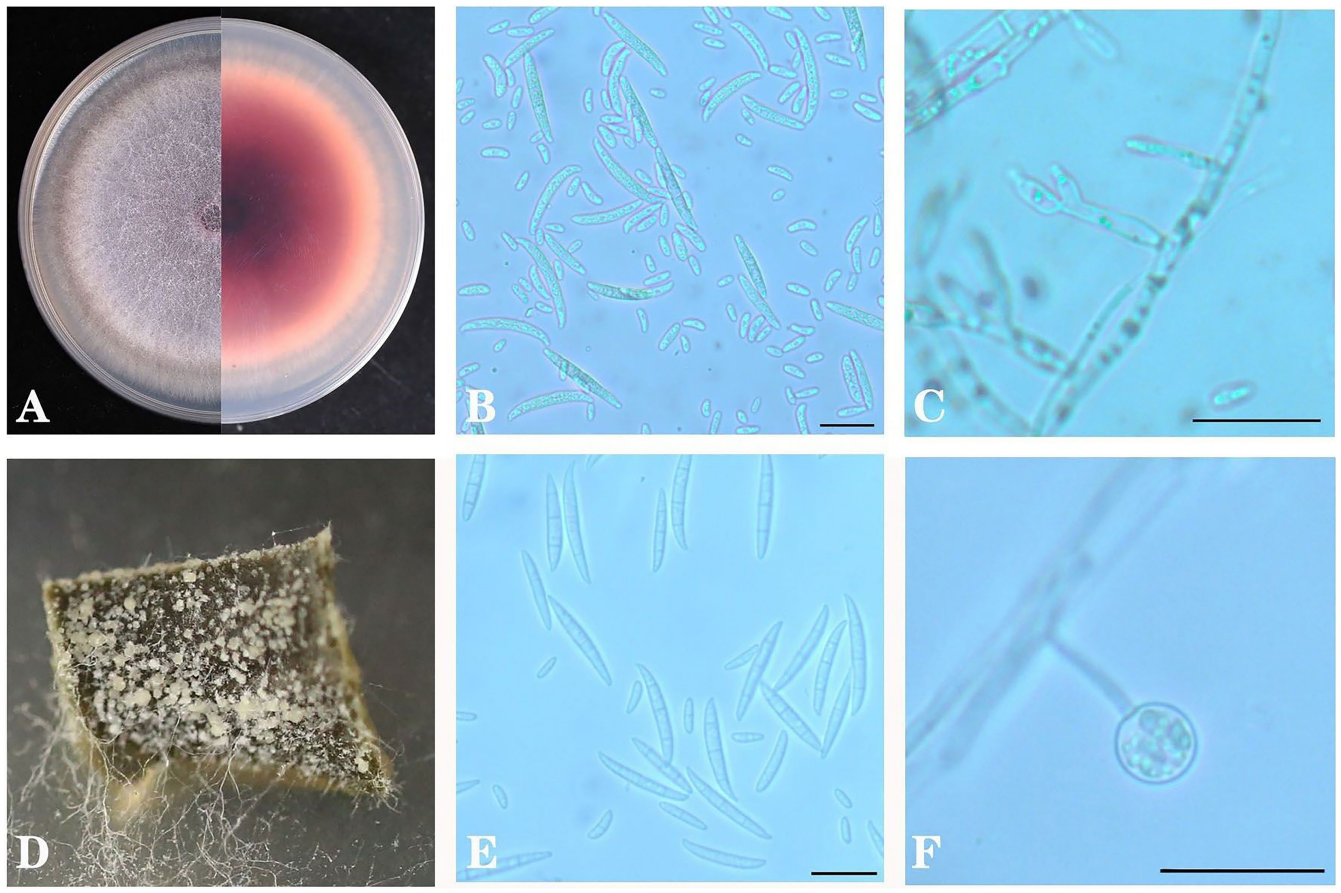
Colony morphology of *Fusarium oxysporum* on PDA **(A)**, conidia **(B)** and conidial fructification **(C)** and on carnation leaf medium **(D)**, conidia **(E)** and conidial fructification **(F)**. Scale bar B = 20 μm in **B,C,E,F**.

After tissue separation and purification, as shown in Figure 8. From the front side of the PDA in all strains, the colony was white to lavender, and it was dark red, with red pigment precipitation from the back side of the plate. Both large and small conidia were observed in the colonies. The small conidia were ovoid or short club-shaped, and they were 6.765–15.902 μm × 1.842–4.313 μm in size. The large conidia were sickle-shaped with one to six septa, and they were 33.691–42.972 μm × 1.844–4.163 μm in size. Moreover, spherical chlamydospores were also observed in the CLA medium. In CLA medium, after 5 days of culture, the white aerial hyphae were flocculent, and the large conidia and chlamydospores were acrogenous.

### Molecular identification and phylogenetic analysis

**Figure 9:**
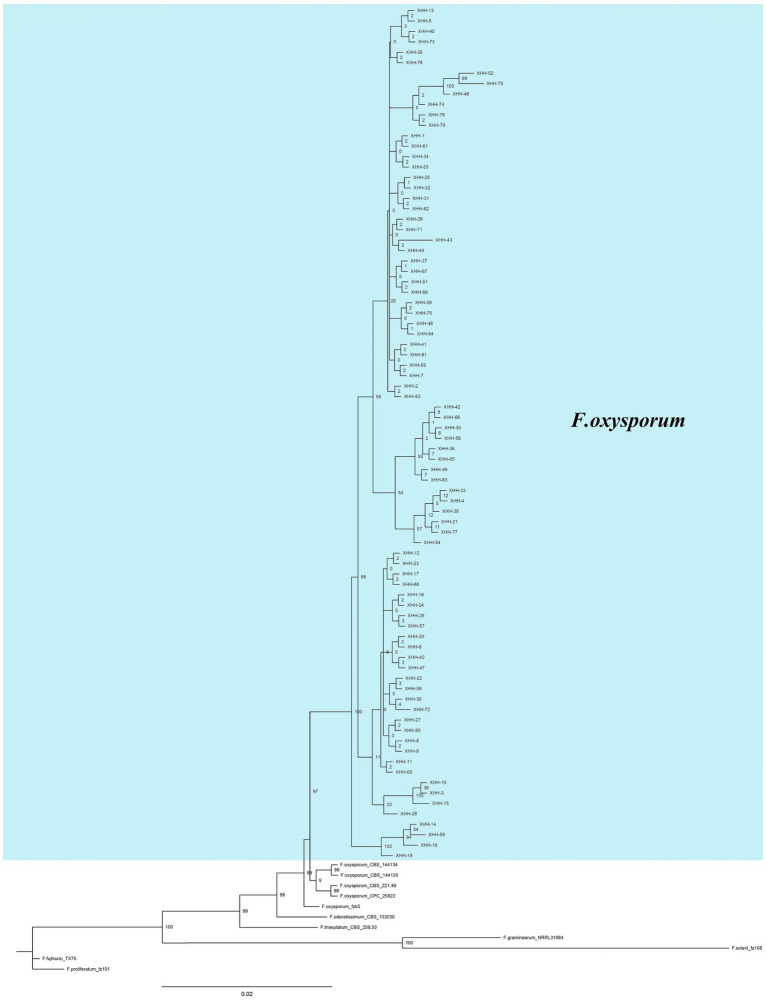
Bayesian inference phylogenetic tree of *Fusarium oxysporum* isolated from saffron corms. The tree was constructed based on ITS, *Tef-1α* and *β-tubulin* genes. *F*. *proliferatum* was used as an outgroup. The scale bar shows 0.02 expected changes per site. XHH represents the number of the strain, the blue part is the strain isolated in this study, others are the strains in the references.

The sequence information was entered into GenBank (Supplementary Table S1). Based on homology comparison results, the ITS sequences were homologous to *F*. *oxysporum*, and the isolates were preliminarily identified as *F*. *oxysporum*. A phylogenetic tree was then established using ITS, *Tef-1α* and *β-tubulin* genes (Figure 9), from which the homology of all isolates with *F*. *oxysporum* was relatively high, reaching 97%. Combined with morphological identification, the strains were further identified as *F*. *oxysporum*.

### Effects of temperature and pH on mycelial growth and sporulation

**Figure 10:**
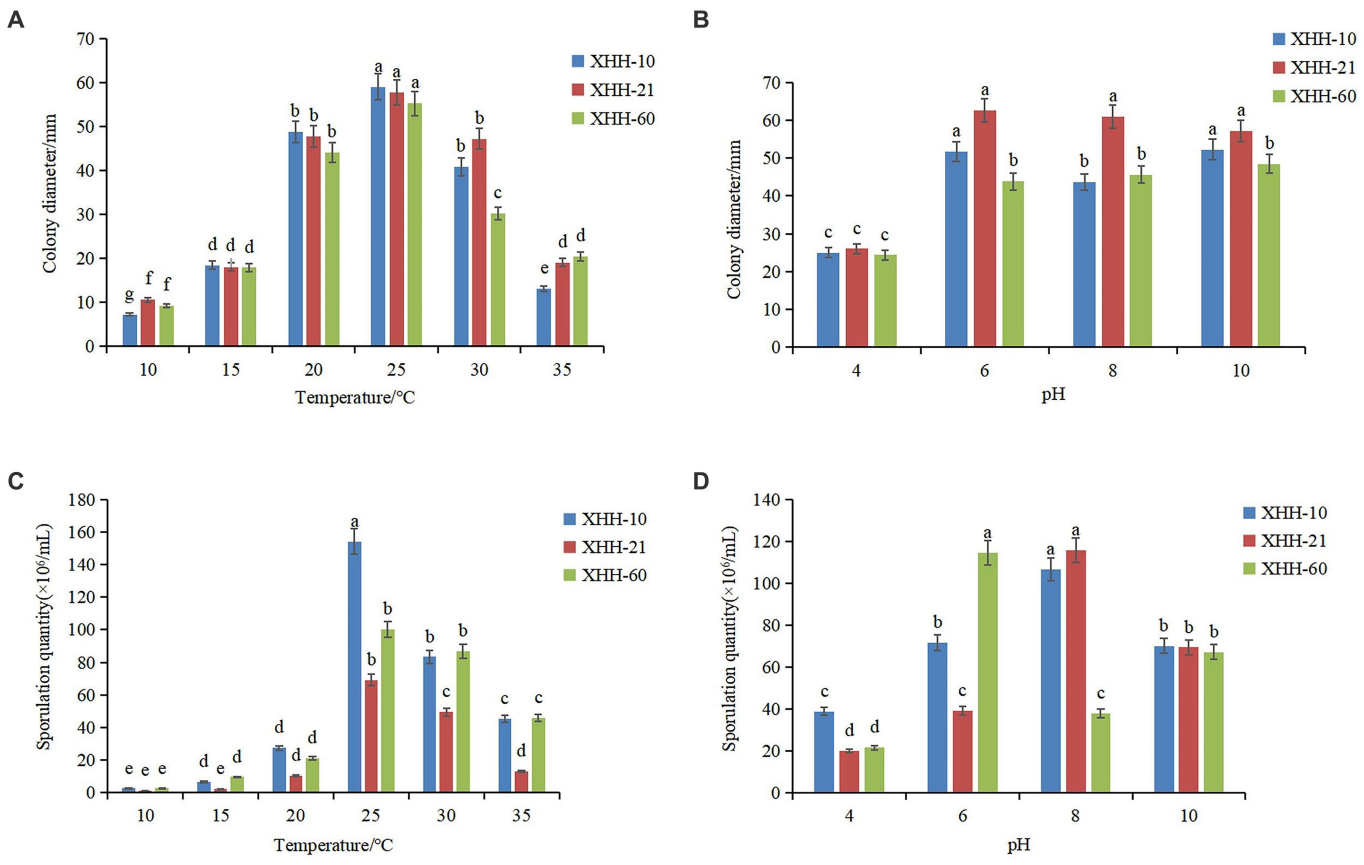
Effects of temperature and pH on colony growth **(A,B)** and sporulation **(C,D)** of *Fusarium oxysporum*. Different lowercase letters indicate a significant difference at the *p* <0.05 level.

*Fusarium oxysporum* can grow in the range of 10°C–35°C (Figure 10A). The mycelial growth rate at 10°C–25°C showed an upward trend, and at 25°C–35°C, it showed a downward trend. It grew fastest at 25°C. The colony diameter was 59.00 mm after 5 days of culture. Similarly, it sporulated in the range of 10°C–35°C and significantly increased at 25°C (Figure 10C). The spore yield of *F*. *oxysporum* at 25°C–30°C was higher. The results showed that the optimum growth temperature of the pathogen was 25°C, and the optimum sporulation temperature was 25°C. Both low- and high-temperature environments had certain inhibitory effects.

*Fusarium oxysporum* can grow under pH 4–10 (Figure 10B). The mycelium grew fastest at pH 6. After 5 days of culture, the colony diameter was 62.67 mm. The sporulation of most *F*. *oxysporum* isolates increased between pH 6 and 10, but the sporulation was highest at pH 8 and lowest at pH 4 (Figure 10D). However, the spore yield of strain XHH-60 increased significantly at pH 6. The optimal growth pH was 6, but the difference was not significant.

### Effects of different carbon and nitrogen sources on growth and sporulation

The pathogen was cultivated on various carbon source media for 5 days, and the growth and sporulation were measured (Table 3). The pathogen grew fastest on sorbitol medium with a diameter of 63.86 ± 0.52 mm, but slowest on maltose medium with a diameter of 39.43 ± 0.41 mm. And low utilization of soluble starch, glucose, fructose, galactose, cellulose and sucrose by pathogen. In addition, the sporulation amount reached the highest on sorbitol medium, but the difference of sporulation amount on other carbon source was small. The pathogen was cultivated on various nitrogen source media for 5 days, and the growth and sporulation were measured meanwhile (Table 4). When peptone and yeast extract were used as nitrogen sources, mycelia grew faster. But when glycine, beef extract, ammonium nitrate, ammonium sulfate and cysteine were used as nitrogen sources, mycelia grew slower. And for sporulation, the highest sporulation quantity was obtained with peptone as nitrogen source, followed by cysteine. The results showed that the utilization rate of peptone was the highest.

**Table 3:** Effects of carbon sources on mycelial growth and sporulation of *Fusarium oxysporum.*

Carbon sources	Colony diameter/mm	Sporulation/10^6^
Starch	52.57 ± 0.98 bc	156.25 ± 0.45 cd
Glucose	56.00 ± 0.41 b	294.00 ± 0.73 bc
Maltone	39.43 ± 0.41 c	263.20 ± 0.81 b
Fructose	57.86 ± 1.03 b	327.50 ± 0.59 b
Galactose	54.57 ± 0.55 bc	209.50 ± 1.01 c
Sorbitol	63.86 ± 0.52 a	505.00 ± 0.92 a
Cellulose	59.43 ± 1.17 b	227.50 ± 0.77 b
Sucrose	55.00 ± 0.89 b	286.00 ± 0.49 bc
No carbon	56.00 ± 0.75 b	76.50 ± 1.17 d

Data are mean ± SD. Different letters in the same column indicate significant difference at *p - globally* < 0.05 level by Duncan’s multiple range test.

**Table 4:** Effects of nitrogen sources on mycelial growth and sporulation of *Fusarium oxysporum.*

Nitrogen sources	Colony diameter/mm	Sporulation/10^6^
Peptone	52.43 ± 0 a	230.00 ± 0.92 a
Yeast extract	53.43 ± 1.17 a	193.50 ± 0.47 b
Glycine	31.00 ± 0 c	142.00 ± 0.73 b
Beef extract	35.00 ± 0.52 c	119.00 ± 0.89 bc
Ammonium nitrate	26.71 ± 0.89 d	153.50 ± 1.42 b
Ammonium sulfate	33.86 ± 0.82 c	177.00 ± 0.84 b
Cysteine	32.14 ± 0.52 c	213.50 ± 0.79 a
No nitrogen	43.29 ± 0.52 b	95.70 ± 1.04 d

Data are mean ± SD. Different letters in the same column indicate significant difference at *P* < 0.05 level by Duncan’s multiple range test.

## Discussion

In recent years, corm rot disease has plagued the development of the saffron industry and caused serious economic losses for growers. However, it is difficult to conduct effective management because of the insufficient understanding of the etiology causing saffron corm rot. Through the amplicon sequencing in this study, we found that the relative abundance of *Fusarium* was lower in healthy plants but significantly increased in rotted corms. And *Penicillium* was the most abundant fungal species in rotted corms, except for those unclassified species. The relative abundance of *Penicillium* significantly increased in rotted corms, suggesting that *Penicillium* might be an opportunistic fungus that coinfects the corms with *Fusarium* or a saprophytic fungus utilizing the nutrition from rotted corms. *Penicillium* is a broad class of fungal microorganisms, most of which are saprophytes. *Penicillium* has a good effect on the biological control of plant diseases, especially soil-borne diseases (Boughalleb et al., 2018; Li et al., 2019; Hassine et al., 2022). We speculated that saffron corm rot caused by *Fusarium* infection occurred before *Penicillium*, accelerating the decay of the corm and having an auxiliary effect. For the soil samples, there was no significant difference in the abundance of *Fusarium* between the two samples, suggesting that the pathogen causing SCR was not derived from the soil and most likely originated from the saffron corm itself. But the pathogens previously reported *Colletotrichum*, *Aspergillus*, *Brasiliensis*, *Rhizoctonia*, *Sclerotinia*, *Phoma*, *Stromatinia*, *Cochliobolus* and *Rhizopus* were not found from saffron corms in this study.

With the decrease in the abundance of *Pseudomonas* in the rotted corms, corm disease became more serious. *Pseudomonas* plays an important role in the development of soil microbial communities rather than pathogenicity (Jatsenko et al., 2010). Root exudate components induce the *Pseudomonas* rhizosphere to form beneficial plant metabolites (Panichikkal and Krishnankutty, 2021). A variety of active metabolites have been found in *Pseudomonas* that can resist harsh environments and many plant diseases (Alam et al., 2022).

By comparing ACE, Chao1, Simpson and Shannon indices in plants and soil, the microbial diversity in rotted corms decreased significantly. At the same time, the microbial diversity of the rotted soil samples also decreased significantly. These results indicate that the disease leads to a decrease in microbial diversity in plants and rhizosphere soil. In the diseased soil samples, Ascomycota increased from 33.72% to 57.86%, while Basidiomycota decreased from 54.63% to 32.63%. At the genus level, *Hypholoma* decreased to 16.75% in the diseased soil. *Botryotinia* was the most abundant species in the diseased soil samples, while it was much lower in healthy soil.

Based on the network diagram, the coexistence of species in the environmental samples was determined. The co-occurrence network analysis is widely used to study the interactions of organisms in complex microbial communities (Faust and Raes, 2021). The co-occurrence network showed that the correlation between fungi and bacterial communities was mainly positive, which maintained the normal growth and development of plants (Gaston et al., 2000). A possible competition between these microbial taxa for soil organic matters, including root secreted photoassimilates (Zhalnina et al., 2018), could explain our findings, with positive correlations dominating microbial communities. The negative correlation between *Oidiodendron* and *Fusarium*, and *Paraphaeosphaeria* and *Fusarium* were observed.

The abundance of *Fusarium* increased, and the abundance of *Oidiodendron* and *Paraphaeosphaeria* decreased. With the development of proteomics and transcriptomics and their application in the study of mycorrhizal fungi, *Oidiodendron maius*, an ericoid mycorrhizal fungi (ErMF) strain that establishes a symbiotic relationship with the blueberry root system, has been shown to promote the growth of blueberry and improve its resistance to disease (Shine et al., 2015; Daghino et al., 2016). The abundance of *Paraphaeosphaeria* increased significantly in the rhizosphere soil fungal community of gentiana planted for 3 years and decreased the abundance of pathogenic fungi *Fusarium* and *Colletotrichum*, which may be of great significance for the prevention and control of gentiana leaf blight (Gao et al., 2021). The FUNGuild fungal phenotype prediction results showed that the soil samples of diseased plants contained fewer nutritive fungi, while the relative abundance of plant pathogens was higher than that of healthy plants.

After traditional isolation culture, 82 isolates were isolated and identified as *F*. *oxysporum* based on morphological and molecular methods and a reinfection experiment, which matched the amplicon sequencing results. The effects of temperature, pH, carbon sources and nitrogen sources on the mycelial growth and sporulation of *F*. *oxysporum* were investigated. The optimal conditions for mycelial growth and sporulation quantity of *F*. *oxysporum* were 25°C and pH 6, sorbitol and peptone.

In a previous research report on saffron corm rot, most *Fusarium* isolates were *F*. *oxysporum* (Michielse and Rep, 2009; Edel-Hermann and Lecomte, 2019). Recently, Mirghasempour et al. (2022a,b) first identified *F. nirenbergiae*, *F. communeas* and *F. annulatum* the predominant agent of corm rot in China, *F. nirenbergiae* was the most. In this study, the causal agent of SCR was identified as *F*. *oxysporum* combining morphological with multilocus (ITS, *Tef*-1α and *β-tubulin*) phylogenetic analysis. Considering the complexity to distinguish the species in *F. oxysporum* species complex, we think more multilocus would facilitate the exact identification of *Fusarium* spp. associated with corm rot. *F. oxysporum* species complex (FOSC) should be a nonnegligible pathogen causing corm rot in the field, and it can infect a variety of economically important plants (Michielse and Rep, 2009; Gao et al., 2021), such as banana (Liu et al., 2019; Tao et al., 2020), watermelon and solanaceae plants, causing serious production losses each year (Borrego-Benjumea et al., 2014; Guo et al., 2021).

Therefore, the combination of amplicon sequencing and traditional isolation culture may reflect the composition and potential pathogens of the rhizosphere microbial community more comprehensively than using these two methods alone, which is of great significance for the prevention and control of saffron disease, whether the disease is associated with pathogenic bacteria and protists (Bahroun et al., 2021) can be observed in the future. Therefore, it is of great significance to explore whether biological agents can be added to prevent and treat saffron corm rot to improve the reproductive capacity of saffron corms and reduce disease occurrence. In addition, soil-borne pathogens need to invade the root microbiome and spread as saprophytes until they reach a trigger population density before causing infection. Thus, pathogen intrusion success is a direct function of its interaction with the rhizosphere microbiome (Wei et al., 2015). If saffron corm rot is a soil-borne disease, water-drought rotation is widely recommended as a cultivation technique for controlling soil-borne diseases (Berendsen et al., 2018). The water-drought rotation cultivation mode in saffron-rice was adopted in saffron-producing areas in Zhejiang Province, but the incidence of corm rot was still serious. This issue remains to be further studied.

## Conclusion

*Fusarium oxysporum* was identified as a pathogen causing saffron corm rot disease by combining the results of traditional isolation and amplicon sequencing in this study. It was further confirmed that the main infection source of the disease was the corms, not soils.

## Data availability statement

The original contributions presented in the study are included in the article/Supplementary material, further inquiries can be directed to the corresponding author.

## Author contributions

TR and TL conceived the study. TR and CZ performed computational analyses and interpreted the data. CZ, TR, DD, and MY wrote and revised the manuscript. All authors contributed to the article and approved the submitted version.

## Funding

This study was supported by grants from “Sannongliufang” Research Joint Project of Zhejiang Province (no. 2021SNLF019) and the Key Research and Development Project of Zhejiang Province, China (no. 2020C02005).

## Conflict of interest

The authors declare that the research was conducted in the absence of any commercial or financial relationships that could be construed as a potential conflict of interest.

## Publisher’s note

All claims expressed in this article are solely those of the authors and do not necessarily represent those of their affiliated organizations, or those of the publisher, the editors and the reviewers. Any product that may be evaluated in this article, or claim that may be made by its manufacturer, is not guaranteed or endorsed by the publisher.

## Footnotes

^1^ http://www.ncbi.nlm.nih.gov/

^2^ https://unite.ut.ee/

## References

[ref2] Ahrazem O. Rubio-Moraga A. Castillo-López R. Mozos A. T. Gómez-Gómez L. (2010). *Crocus sativus* pathogens and defense responses. Func. Plant Sci. Biotec., 13, 297–303. doi: 10.1111/j.1438-8677.2010.00359.x

[ref3] Alam K. M. Yan Y. L. Lin M. Islam M. A. Gaber A. Hossain A. (2022). Insight rifampicin-resistant (rpoB) mutation in *Pseudomonas stutzeri* leads to enhance the biosynthesis of secondary metabolites to survive against harsh environments. Arch. Microbiol. 204:437. doi: 10.1007/S00203-022-03064-9, PMID: 35768665

[ref4] Amata R. L. Burgess L. W. Summerell B. A. Bullock S. Liew E. C. Y. Smith-White J. L. (2010). An emended description of *Fusarium brevicatenulatum* and *F. pseudoanthophilum* based on isolates recovered from millet in Kenya. Fungal Divers. 43, 11–25. doi: 10.1007/s13225-010-0019-3

[ref5] Bahrami A. Mirmohammadsadegh S. S. Zare-Hoseini S. F. Mousavi-Tabari R. Saket S. (2020). Saffron in the ancient history of Iran. Saffron. 3, 23–34. doi: 10.1016/B978-0-12-818638-1.00003-4

[ref6] Bahroun A. Jousset A. Mrabet M. Mhamdi R. (2021). Protists modulate *Fusarium* root rot suppression by beneficial bacteria. Appl. Soil Ecol. 168:104158. doi: 10.1016/j.apsoil.2021.104158

[ref7] Berendsen R. L. Vismans G. Yu K. Song Y. Jonge R. Burgman W. P.. (2018). Disease-induced assemblage of a plant-beneficial bacterial consortium. ISME J. 12, 1496–1507. doi: 10.1038/s41396-018-0093-1, PMID: 29520025 PMC5956071

[ref8] Bokulich N. A. Subramanian S. Faith J. J. Gevers D. Gordon J. I. Knight R.. (2013). Quality-filtering vastly improves diversity estimates from Illumina amplicon sequencing. Nat. Methods 10, 57–59. doi: 10.1038/nmeth.2276, PMID: 23202435 PMC3531572

[ref9] Borrego-Benjumea A. Basallote-Ureba M. J. Melero-Vara J. M. Abbasi P. A. (2014). Characterization of *fusarium* isolates from asparagus fields in southwestern Ontario and influence of soil organic amendments on *Fusarium* crown and root rot. Phytopathology 104, 403–415. doi: 10.1094/PHYTO-08-13-0231-R, PMID: 24261409

[ref10] Boughalleb M. N. Salem I. B. Mahmoud M. H. (2018). Evaluation of the efficiency of *Trichoderma*, *Penicillium*, and *Aspergillus* species as biological control agents against four soil-borne fungi of melon and watermelon. Egypt J Biol Pest Co. 28, 1–12. doi: 10.1186/s41938-017-0010-3

[ref11] Britz H. Wingfield M. J. Coutinho T. A. Marasas W. F. O. Leslie J. F. (1998). Female fertility and mating type distribution in a c population of *Fusarium subglutinans* f.sp. *pini*. Appl. Environ. Microbiol. 64, 2094–2095. doi: 10.1128/AEM.64.6.2094-2095.1998, PMID: 9603819 PMC106283

[ref12] Cai L. Hyde K. D. Taylor P. Weir B. S. Waller J. M. Abang M. M.. (2009). A polyphasic approach for studying *Colletotrichum*. Fungal Divers. 26, 252–254. doi: 10.1016/j.riam.2009.11.001

[ref14] Chen Q. (2018). Successful declaration of geographical indication of national agricultural products by *Crocus sativus* in Jiande city. Hangzhou 33:62. doi: 10.16639/j.cnki.cn33-1361/d.2018.33.016

[ref15] Chen X. Y. Dai D. J. Zhao S. F. Shen Y. Wang H. D. Zhang C. Q. (2020). Genetic diversity of *Colletotrichum* spp. causing strawberry anthracnose in Zhejiang, China. Plant Dis. 104, 1351–1357. doi: 10.1094/PDIS-09-19-2026-RE, PMID: 32213124

[ref16] Chen F. Ma R. Chen X. L. (2019). Advances of metabolomics in fungal pathogen-plant interactions. Meta 9, 169. doi: 10.3390/metabo9080169 PMC6724083 31443304

[ref17] Crous P. W. Lombard L. Sandoval-Denis M. Seifert K. A. Schroers H. J. Chaverri P.. (2021). *Fusarium*: more than a node or a foot-shaped basal cell. Stud Mycol. 98:100116. doi: 10.1016/j.simyco.2021.100116 34466168 PMC8379525

[ref18] Daghino S. Martino E. Perotto S. (2016). Model systems to unravel the molecular mechanisms of heavy metal tolerance in the ericoid mycorrhizal symbiosis. Mycorrhiza 26, 263–274. doi: 10.1007/s00572-015-0675-y 26710764

[ref19] Deng S. F. Wang X. R. Zhang A. C. Chen C. E. Zhou L. (2019). Study on *Crocus sativus*—rice rotation pattern in the middle and lower reaches of Yangtze River. Jiangxi Agric. 14, 7–10. doi: 10.19394/j.cnki.issn1674-4179.2019.14.006

[ref20] Dissanayake A. J. Purahong W. Wubet T. Hyde K. D. Zhang W. Xu H.. (2018). Direct comparison of culture-dependent and culture-independent molecular approaches reveal the diversity of fungal endophytic communities in stems of grapevine (*Vitis vinifera*). Fungal Divers. 90, 85–107. doi: 10.1007/s13225-018-0399-3

[ref21] Edel-Hermann V. Lecomte C. (2019). Current status of *Fusarium oxysporum* formae speciales and races. Phytopathology 109, 512–530. doi: 10.1094/PHYTO-08-18-0320-RVW, PMID: 30461350

[ref22] Faust K. Raes J. (2021). Microbial interactions: from networks to models. Nat. Rev. Microbiol. 10, 538–550. doi: 10.1038/nrmicro2832 22796884

[ref23] Gale L. R. Bryant J. D. Calvo S. Giese H. Katan T. O'Donnell K.. (2005). Chromosome complement of the fungal plant pathogen *Fusarium graminearum* based on genetic and physical mapping and cytological observations. Genetics 171, 985–1001. doi: 10.1534/genetics.105.044842, PMID: 16079234 PMC1456848

[ref24] Gao S. Sun W. S. Yu C. L. Li L. Zhang T. J. (2021). Analysis of soil fungal community structure in gentiana rhizosphere based on high-throughput sequencing. Jiangsu Agric. Sci. 49, 190–195. doi: 10.15889/j.issn.1002-1302.2021.12.032

[ref25] Gao M. Xiong C. Gao C. Clement K. M. Wang M. M. Zhou X.. (2021). Disease-induced changes in plant microbiome assembly and functional adaptation. Microbiome 9:187. doi: 10.1186/S40168-021-01138-2, PMID: 34526096 PMC8444440

[ref26] Gaston K. J. Blackburn T. M. Greenwood J. J. D. Gregory R. D. Quinn R. M. Lawton J. H. (2000). Abundance-occupancy relationships. J. Appl. Ecol. 37, 39–59. doi: 10.1046/j.1365-2664.2000.00485.x

[ref27] Grady K. L. Sorensen J. W. Stopnisek N. Guittar J. Shade A. (2019). Assembly and seasonality of core phyllosphere microbiota on perennial biofuel crops. Nat. Commun. 10:4135. doi: 10.1101/446369, PMID: 31515535 PMC6742659

[ref28] Gu Y. Banerjee S. Dini Andreote F. Xu Y. C. Shen Q. R. Jousset A.. (2022). Small changes in rhizosphere microbiome composition predict disease outcomes earlier than pathogen density variations. ISME J. 16, 2448–2456. doi: 10.1038/S41396-022-01290-Z, PMID: 35869387 PMC9478146

[ref29] Guo Z. N. Yu Z. H. Li Q. L. Tang L. H. Guo T. X. Huang S. P.. (2021). *Fusarium* species associated with leaf spots of mango in China. Microb. Pathog. 150:104736. doi: 10.1016/j.micpath.2021.104736, PMID: 33453315

[ref30] Hassine M. Aydi B. A. R. Jabnoun K. H. Daami R. M. (2022). Soil-borne and compost-borne *Penicillium* sp. and *Gliocladium* spp. as potential microbial biocontrol agents for the suppression of anthracnose-induced decay on tomato fruits. Egypt J Biol Pest Co. 32, 1–12. doi: 10.1186/S41938-022-00519-5

[ref31] Hu S. Wang X. X. Sun W. J. Wang L. L. Li W. K. (2021). Investigation on main disease types of *Crocus sativus* in Chongming. Chin. Soc. Plant Pathol. 1, 148–178. doi: 10.26914/c.cnkihy.2021.063781

[ref32] Hu S. D. Zhang Y. T. Yu H. Zhou J. Y. Hu M. H. Liu A. C.. (2022). *Colletotrichum Spp.* diversity between leaf anthracnose and crown rot from the same strawberry plant. Front. Microbiol. 13:860694. doi: 10.3389/FMICB.2022.860694 35495690 PMC9048825

[ref33] Huo J. H. Wen X. L. Li S. M. Fen L. N. Lan S. H. Dong L. X.. (2022). Identification and biological characteristics of root rot of *Atractylodis rhizoma*. China Agric. Sci. Tech. Rep. 24, 137–144. doi: 10.13304/j.nykjdb.2021.0468

[ref34] Jatsenko T. Tover A. Tegova R. Kivisaar M. (2010). Molecular characterization of Rifr mutations in *Pseudomonas aeruginosa* and *Pseudomonas putida*. Mutat. Res. 683, 106–114. doi: 10.1016/j.mrfmmm.2009.10.015 19887074

[ref35] Jia M. Jia L. M. Li N. B. Ma Z. J. Ma J. (2022). Crocin regulates hexokinase and promotes glycolysis to improve myocardial ischemia-reperfusion injury. Hebei Pharm. 44, 1663–1666. doi: 10.3969/j.issn.1002-7386.2022

[ref36] Krzyśko-Lupicka T. Strof W. Kubś K. Skorupa M. Wieczorek P. Lejczak B.. (1997). The ability of soil-borne fungi to degrade organophosphonate carbon-to-phosphorus bonds. Appl. Microbiol. Biotechnol. 48, 549–552. doi: 10.1007/s002530051095, PMID: 9390463

[ref37] Laforest-Lapointe I. Paquette A. Messier C. Kembel S. W. (2017). Leaf bacterial diversity mediates plant diversity and ecosystem function relationships. Nature 546, 145–147. doi: 10.1038/nature22399, PMID: 28538736

[ref38] Li Y. Guo Q. Wei X. Xue Q. Lai H. (2019). Biocontrol effects of *Penicillium* griseofulvum against monkshood (*Aconitum carmichaelii* Debx.) root diseases caused by *Sclerotium rolfsiii* and *Fusarium* spp. J. Appl. Microbiol. 127, 1532–1545. doi: 10.1111/jam.14382, PMID: 31304623

[ref39] Li C. Q. Li J. Q. Wang X. C. Niu Y. L. Qu J. R. (2022). Isolation, identification and biological characteristics of pathogens causing root rot of *Ricinus communis L*. Acta Pratacul. Sin. 31, 113–123.

[ref40] Liu B. B. Dong Y. Yao C. Jiang F. Q. (2022). The resource development of *Crocus sativus* in China studies general situation. Mod. Appl. Sci. Chin., 39, 1783–1788. doi: 10.13748/j.cnki.issn1007-7693.2022.13.0191-6

[ref41] Liu H. W. Li J. Y. Carvalhais L. C. Percy C. D. Prakash V. J. Schenk P. M.. (2021). Evidence for the plant recruitment of beneficial microbes to suppress soilborne pathogens. New Phytol. 229, 2873–2885. doi: 10.1111/nph.17057, PMID: 33131088

[ref42] Liu Y. P. Zhu A. P. Tan H. M. Cao L. X. Zhang R. D. (2019). Engineering banana endosphere microbiome to improve *Fusarium* wilt resistance in banana. Microbiome 7, 1–15. doi: 10.1186/s40168-019-0690-x, PMID: 31092296 PMC6521393

[ref43] Lu J. Breitwieser F. P. Thielen P. Salzberg S. L. (2017). Bracken: estimating species abundance in metagenomics data. Peer J Comp Sci. 3:e104. doi: 10.7717/peerj-cs.104 PMC12016282 40271438

[ref44] Luo L. Y. Zhang Z. Wang P. Han Y. Q. Jin D. C. Su P.. (2019). Variations in phyllosphere microbial community along with the development of angular leaf-spot of cucumber. AMB Express 9, 76–13. doi: 10.1186/s13568-019-0800-y, PMID: 31134393 PMC6536563

[ref45] Masenya K. Thompson G. D. Tekere M. Makhalanyane T. P. Pierneef R. E. Rees D. J. G. (2021). Pathogen infection influences a distinct microbial community composition in sorghum RILs. Plant and Soil 463, 555–572. doi: 10.1007/s11104-021-04875-3

[ref46] Michielse C. B. Rep M. (2009). Pathogen profile update: *Fusarium oxysporum*. Mol. Plant Pathol. 10, 311–324. doi: 10.1111/j.1364-3703.2009.00538.x 19400835 PMC6640313

[ref47] Mirghasempour S. A. Studholme D. J. Chen W. L. Cui D. Mao B. (2022a). Identification and characterization of *Fusarium nirenbergiae* associated with saffron corm rot disease. Plant Dis. 106, 486–495. doi: 10.1094/PDIS-04-21-0871-RE, PMID: 35113681

[ref48] Mirghasempour S. A. Studholme D. J. Chen W. L. Zhu W. Mao B. (2022b). Molecular and pathogenic characterization of *Fusarium* species associated with corm rot disease in saffron from China. J fungi 8:515. doi: 10.3390/jof8050515, PMID: 35628770 PMC9147734

[ref49] Nguyen N. H. Song Z. Bates S. T. Branco S. Tedersoo L. Menke J.. (2016). FUNGuild: an open annotation tool for parsing fungal community datasets by ecological guild. Fungal Ecol. 20, 241–248. doi: 10.1016/j.funeco.2015.06.006

[ref50] Noman E. Al-Gheethi A. A. Rahman N. K. Talip B. Mohamed R. Kadir O. A. (2018). Single spore isolation as a simple and efficient technique to obtain fungal pure culture. Earth Environ. Sci. 140:012055. doi: 10.1088/1755-1315/140/1/012055

[ref51] O’Donnell K. Deanna A. S. Michael G. R. Brice A. J. S. Balajee S. A. Hans Josef S.. (2010). Internet-accessible DNA sequence database for identifying fusaria from human and animal infections. J. Clin. Microbiol. 48, 3708–3718. doi: 10.1128/JCM.00989-10, PMID: 20686083 PMC2953079

[ref52] Paloma D. Thorsten T. Ruben G. Matthew A. Eric K. Paul S. L.. (2018). Microbial interkingdom interactions in roots promote Arabidopsis survival. Cells 175, 973–983.e14. doi: 10.1016/j.cell.2018.10.020, PMID: 30388454 PMC6218654

[ref53] Panichikkal J. Krishnankutty R. E. (2021). Root exudate components induced formation of plant beneficial metabolites by rhizospheric *Pseudomonas* spp. Rhizosphere. 19, 100366–100368. doi: 10.1016/J.RHISPH.2021.100366

[ref54] Paulson J. N. Stine O. C. Bravo H. C. Pop M. (2013). Differential abundance analysis for microbial marker-gene surveys. Nat. Methods 10, 1200–1202. doi: 10.1038/nmeth.2658 24076764 PMC4010126

[ref55] Phoulivong S. Cai L. Chen H. Mckenzie E. H. C. Abdelsalam K. Chukeatirote E. (2010). *Colletotrichum gloeosporioides* is not a common pathogen on tropical fruits. Fungal Divers. 44, 33–43. doi: 10.1007/s13225-0100046-0

[ref56] Qiu R. Li Q. Li J. Dong N. Y. Li S. J. Guan W. D.. (2021). First report of *Fusarium* root rot of tobacco caused by *Fusarium sinensis* in Henan province China. Plant Dis., 3305–3306. doi: 10.1094/PDIS-11-20-2466-PDN, PMID: 33754853

[ref57] Rahjoo V. Zad J. Javan-Nikkhah M. Gohari A. M. Okhovvat S. M. Bihamta M. R.. (2008). Morphological and molecular identification of *Fusarium* isolated from maize ears in Iran. J. Plant Pathol. 110, 7–14. doi: 10.1016/S0169-328X(02)00544-2

[ref58] Ren Q. X. Zhang J. X. Wang J. H. Xu S. Q. Yao W. Zhang M. Q. (2022). Isolation, identification and biological characteristics analysis of pathogen causing sugarcane root rot. Shandong Agric. Sci., 54, 135–142. doi: 10.14083/j.issn.1001-4942.2022.07.019

[ref59] Shine A. M. Shakya V. P. Idnurm A. (2015). Phytochelatin synthase is required for tolerating metal toxicity in a basidiomycete yeast and is a conserved factor involved in metal homeostasis in fungi. Fung. Biol. Biotec. 2, 3–7. doi: 10.1186/s40694-015-0013-3, PMID: 25926993 PMC4410428

[ref60] Stone B. W. Weingarten E. A. Jackson C. R. (2018). The role of the phyllosphere microbiome in plant health and function. Annu. Plant Rev. Onl. 1, 533–556. doi: 10.1002/9781119312994.apr0614

[ref61] Tao C. Y. Li R. Xiong W. Shen Z. Z. Liu S. S. Wang B. B.. (2020). Bio-organic fertilizers stimulate indigenous soil *Pseudomonas* populations to enhance plant disease suppression. Microbiome 8:137. doi: 10.21203/rs.3.rs-18216/v1, PMID: 32962766 PMC7510105

[ref62] Wang J. Gan L. Y. Ma F. F. Zhang S. H. (2021). Efficient *Crocus sativus*—single cropping rice rotation. New Rur. Tec. 4, 12–13. doi: 10.15904/j.cnki.hnny.2020.31.050

[ref63] Wang L. Zhi H. H. Ma Y. Q. Chen H. Y. Zhang G. Guo Q. Y. (2022). Biological characteristics of cherry leaf spot and screening of indoor agents. Fujian J Agric Sci. 37, 503–513. doi: 10.19303/j.issn.1008-0384.2022.004.011

[ref64] Wei L. Duan X. M. Lu G. X. Chang J. P. Zhou X. J. Ma H. X.. (2021). Pathogen identification and indoor screening of control agents for corm rot of saffron. Plant Prot. 47, 139–145. doi: 10.16688/j.zwbh.2020521

[ref65] Wei Y. J. Wu Y. Yan Y. Z. Zou W. Xue J. Ma W. R.. (2018). High-throughput sequencing of microbial community diversity in soil, grapes, leaves, grape juice and wine of grapevine from China. PLoS One 13:e0193097. doi: 10.1371/journal.pone.0193097, PMID: 29565999 PMC5863948

[ref66] Wei Z. Yang T. Friman V.-P. Xu Y. Shen Q. Jousset A. (2015). Trophic network architecture of root-associated bacterial communities determines pathogen invasion and plant health. Nat. Commun. 6:8413. doi: 10.1038/ncomms9413, PMID: 26400552 PMC4598729

[ref67] Xiang L. G. Wang H. C. Wang F. Cai L. T. Li W. H. Hsiang T.. (2022). Analysis of phyllosphere microorganisms and potential pathogens of tobacco leaves. Front. Microbiol. 13:843389. doi: 10.3389/FMICB.2022.843389, PMID: 35572673 PMC9100574

[ref68] Zhalnina K. Louie K. B. Hao Z. Mansoori N. Rocha U. N. Shi S.. (2018). Dynamic root exudate chemistry and microbial substrate preferences drive patterns in rhizosphere microbial community assembly. Nat. Microbiol. 3, 470–480. doi: 10.1038/s41564-018-0129-3, PMID: 29556109

[ref70] Zhang G. H. Zhang X. P. Zhang N. F. He D. Y. (2009). Pathogen identification and drug prevention of *Crocus sativus* corm rot. J. Kaili Univ. 27, 47–49. doi: 10.3969/j.issn.1673-9329.2009.03.019

[ref71] Zhao L. J. (2014). Isolation and identification of pathogenic bacteria from saffron and screening of its preventive and therapeutic drugs. Shanghai Norm. Univ., 23–35.

[ref72] Zhou J. Chen Q. H. Xu J. W. Chen H. Huang B. S. Miao Y. H.. (2022). Pathogen identification, biological characteristics determination and control agents screening of white silk disease. Chin. J Chin. Mat. Medi. 47, 5209-5216. doi: 10.19540/j.cnki.cjcmm.20220615.103

