# Supplementary material for: Identification and characterization of pathogens causing saffron corm rot in China
Source: Front Microbiol. 2023 Jun 9;14:1188376. doi: 10.3389/fmicb.2023.1188376 (PMC10289022; doi:10.3389/fmicb.2023.1188376)
Supplement: Supplementary file 1 [file Table_1.pdf]

Table S1. Fungi isolated from rotted corms, and their identity based on ITS, Translation elongation factor 1-α and *β-tubulin* sequences, and their GenBank accession numbers

Species	Strain name	GenBank Accession Number		
		ITS	Translation elongation factor 1-α	β-tubulin
Fusarium oxysporum	XHH1	ON878280	ON970746	ON970829
Fusarium oxysporum	XHH2	ON878281	ON970757	ON970830
Fusarium oxysporum	XHH3	ON878282	ON970768	ON970831
Fusarium oxysporum	XHH4	ON878283	ON970779	ON970832
Fusarium oxysporum	XHH5	ON878284	ON970790	ON970833
Fusarium oxysporum	XHH6	ON878285	ON970801	ON970834
Fusarium oxysporum	XHH7	ON878286	ON970812	ON970835
Fusarium oxysporum	XHH8	ON878287	ON970823	ON970836
Fusarium oxysporum	XHH9	ON878288	ON970827	ON970837
Fusarium oxysporum	XHH10	ON878289	ON970747	ON970838
Fusarium oxysporum	XHH11	ON878290	ON970748	ON970839
Fusarium oxysporum	XHH12	ON878291	ON970749	ON970840
Fusarium oxysporum	XHH13	ON878292	ON970750	ON970841
Fusarium oxysporum	XHH14	ON878293	ON970751	ON970842
Fusarium oxysporum	XHH15	ON878294	ON970752	ON970843
Fusarium oxysporum	XHH16	ON878295	ON970753	ON970844
Fusarium oxysporum	XHH17	ON878296	ON970754	ON970845
Fusarium oxysporum	XHH18	ON878297	ON970755	ON970846
Fusarium oxysporum	XHH19	ON878298	ON970756	ON970847
Fusarium oxysporum	XHH20	ON878299	ON970758	ON970848
Fusarium oxysporum	XHH21	ON878300	ON970759	ON970849
Fusarium oxysporum	XHH22	ON878301	ON970760	ON970850
Fusarium oxysporum	XHH23	ON878302	ON970761	ON970851
Fusarium oxysporum	XHH24	ON878303	ON970762	ON970852
Fusarium oxysporum	XHH25	ON878304	ON970763	ON970853
Fusarium oxysporum	XHH26	ON878305	ON970764	ON970854
Fusarium oxysporum	XHH27	ON878306	ON970765	ON970855
Fusarium oxysporum	XHH28	ON878307	ON970766	ON970856
Fusarium oxysporum	XHH29	ON878308	ON970767	ON970857
Fusarium oxysporum	XHH30	ON878309	ON970769	ON970858
Fusarium oxysporum	XHH31	ON878310	ON970770	ON970859
Fusarium oxysporum	XHH32	ON878311	ON970771	ON970860
Fusarium oxysporum	XHH33	ON878312	ON970772	ON970861
Fusarium oxysporum	XHH34	ON878313	ON970773	ON970862
Fusarium oxysporum	XHH35	ON878314	ON970774	ON970863
Fusarium oxysporum	XHH36	ON878315	ON970775	ON970864
Fusarium oxysporum	XHH37	ON878316	ON970776	ON970865
Fusarium oxysporum	XHH38	ON878317	ON970777	ON970866

Fusarium oxysporum	XHH39	ON878318	ON970778	ON970867
Fusarium oxysporum	XHH40	ON878319	ON970780	ON970868
Fusarium oxysporum	XHH41	ON878320	ON970781	ON970869
Fusarium oxysporum	XHH42	ON878321	ON970782	ON970870
Fusarium oxysporum	XHH43	ON878322	ON970783	ON970871
Fusarium oxysporum	XHH44	ON878323	ON970784	ON970872
Fusarium oxysporum	XHH45	ON878324	ON970785	ON970873
Fusarium oxysporum	XHH46	ON878325	ON970786	ON970874
Fusarium oxysporum	XHH47	ON878326	ON970787	ON970875
Fusarium oxysporum	XHH48	ON878327	ON970788	ON970876
Fusarium oxysporum	XHH49	ON878328	ON970789	ON970877
Fusarium oxysporum	XHH50	ON878329	ON970791	ON970878
Fusarium oxysporum	XHH51	ON878330	ON970792	ON970879
Fusarium oxysporum	XHH52	ON878331	ON970793	ON970880
Fusarium oxysporum	XHH53	ON878332	ON970794	ON970881
Fusarium oxysporum	XHH54	ON878333	ON970795	ON970882
Fusarium oxysporum	XHH55	ON878334	ON970796	ON970883
Fusarium oxysporum	XHH56	ON878335	ON970797	ON970884
Fusarium oxysporum	XHH57	ON878336	ON970798	ON970885
Fusarium oxysporum	XHH58	ON878337	ON970799	ON970886
Fusarium oxysporum	XHH59	ON878338	ON970800	ON970887
Fusarium oxysporum	XHH60	ON878339	ON970802	ON970888
Fusarium oxysporum	XHH61	ON878340	ON970803	ON970889
Fusarium oxysporum	XHH62	ON878341	ON970804	ON970890
Fusarium oxysporum	XHH63	ON878342	ON970805	ON970891
Fusarium oxysporum	XHH64	ON878343	ON970806	ON970892
Fusarium oxysporum	XHH65	ON878344	ON970807	ON970893
Fusarium oxysporum	XHH66	ON878345	ON970808	ON970894
Fusarium oxysporum	XHH67	ON878346	ON970809	ON970895
Fusarium oxysporum	XHH68	ON878347	ON970810	ON970896
Fusarium oxysporum	XHH69	ON878348	ON970811	ON970897
Fusarium oxysporum	XHH70	ON878349	ON970813	ON970898
Fusarium oxysporum	XHH71	ON878350	ON970814	ON970899
Fusarium oxysporum	XHH72	ON878351	ON970815	ON970900
Fusarium oxysporum	XHH73	ON878352	ON970816	ON970901
Fusarium oxysporum	XHH74	ON878353	ON970817	ON970902
Fusarium oxysporum	XHH75	ON878354	ON970818	ON970903
Fusarium oxysporum	XHH76	ON878355	ON970819	ON970904
Fusarium oxysporum	XHH77	ON878356	ON970820	ON970905
Fusarium oxysporum	XHH78	ON878357	ON970821	ON970906
Fusarium oxysporum	XHH79	ON878358	ON970822	ON970907
Fusarium oxysporum	XHH80	ON878359	ON970824	ON970908
Fusarium oxysporum	XHH81	ON878360	ON970825	ON970909
Fusarium oxysporum	XHH82	ON878361	ON970826	ON970910

Fusarium oxysporum	CBS144134	MT997878	MH485044	NW022158520
Fusarium oxysporum	CBS144135	MN709613	MH485045	NW022158693
Fusarium oxysporum	CBS221.49	DQ453704	MH484963	NW022158730
Fusarium oxysporum	CPC25822	KT794176	MH485034	NC030990
Fusarium oxysporum	fzk5	AY928412	MT723973	U34424
Fusarium odoratissimum	CBS102030	MW016596	MH484989	MW533954
Fusarium triseptatum	CBS258.50	MW016606	MH484964	MW533960
Fusarium graminearum	NRRL31084	DQ459823	MH572253	NC026477
Fusarium solani	fz108	KY910890	MT723976	NW025764830
Fusarium fujkuroi	TX75	DQ094534	MH828026	MH827999
Fusarium proliferatum	fz101	MF567504	MT723967	KC571341
